# Predicting mortality and visualizing health care spending by predicted mortality in Danes over age 65

**DOI:** 10.1038/s41598-023-28102-4

**Published:** 2023-01-21

**Authors:** Anne Vinkel Hansen, Laust Hvas Mortensen, Claus Thorn Ekstrøm, Stella Trompet, Rudi Westendorp

**Affiliations:** 1 Methods and Analysis, Statistics Denmark, , Danmarks Statistik, Sejrøgade 11, 2100 Copenhagen, Denmark; 2 Center for Healthy Aging, University of Copenhagen, Copenhagen, Denmark; 3 Section of Epidemiology, Department of Public Health, University of Copenhagen, Copenhagen, Denmark; 4 Section of Biostatistics, Department of Public Health, University of Copenhagen, Copenhagen, Denmark; 5 Department of Internal Medicine, Section of Gerontology and Geriatrics, Leiden University Medical Center, Leiden, the Netherlands

**Keywords:** Health care economics, Epidemiology

## Abstract

Health care expenditure in the last year of life makes up a high proportion of medical spending across the world. This is often framed as waste, but this framing is only meaningful if it is known at the time of treatment who will go on to die. We analyze the distribution of health care spending by predicted mortality for the Danish population over age 65 over the year 2016, with one-year mortality predicted by a machine learning model based on sociodemographics and use of health care services for the two years before entry into follow-up. While a reasonably good model can be built, extremely few individuals have high ex-ante probability of dying, and those with a predicted mortality of more than 50% account for only 2.8% of total health care expenditure. Decedents outspent survivors by a factor of more than ten, but compared to survivors with similar predicted mortality they spent only 2.5 times as much. Our results suggest that while spending in the last year of life is indeed high, this is nearly all spent in situations where there is a reasonable expectation that the patient can survive.

## Introduction

Healthcare expenditure in the last year of life—the “cost of dying”—accounts for between 8.5 and 11.2%^1^ of total medical spending. This is even more pronounced at higher ages since this is where mortality is concentrated. A reduction of this expense is an alluring prospect as it would not only reduce the economic burden of an ageing population but potentially also save people useless treatment^2–5^.

However, the assertion that we spend a lot on those who die is misleading: We can only talk of money wasted on those who go on to die if we can know at the time of starting treatment that the patient will die within a short enough time frame to make treatment frivolous. Recent applications of machine learning to improve prediction of mortality have shown impressive results^6,7^. Nonetheless, mortality seems to remain to a large extent stochastic^8,9^—even at very high ages, there is still a substantial probability of living to see another birthday. In fact, the discriminative ability of a number of ‘classic’ health indicators seems to decline with age^10^. In a 2018 paper^2^, Einav et al. showed that even a well-performing, state-of-the-art prediction model when predicting one-year mortality for a representative sample of American Medicare enrollees finds very few individuals with more than a 50% risk of dying within a year. It is only with a very high mortality prediction in hand that one could consider to refrain from initiating medical treatment but due to their rarity, individuals with very high mortality risk make up a negligible share of population healthcare expenditure, even though their individual healthcare expenses are high^2^.

In this paper, we attempt to reproduce the analyses of Einav et al.^2^ and explore the distribution of healthcare expenditure by predicted mortality in the Danish population over age 65, using state-of-the-art machine learning methods and the rich national registry data to obtain the best possible prediction of mortality. With this exercise we will explore whether the patterns of mortality and health care expenditure as described for American Medicare enrollees can be found in a different population (covering an entire country) and healthcare system. Next, we explore whether the inclusion of a wider range of socioeconomic variables that are present in the Danish national registries will improve prediction to any material extent, and how the inclusion of the costs of communal care affects the conclusions. The costs of communal care are often ignored when discussing the cost of dying, but these make up a sizeable proportion of the actual societal costs of dying, and the proportion grows with the age of decedents^11^.

## Results

**Figure 1:**
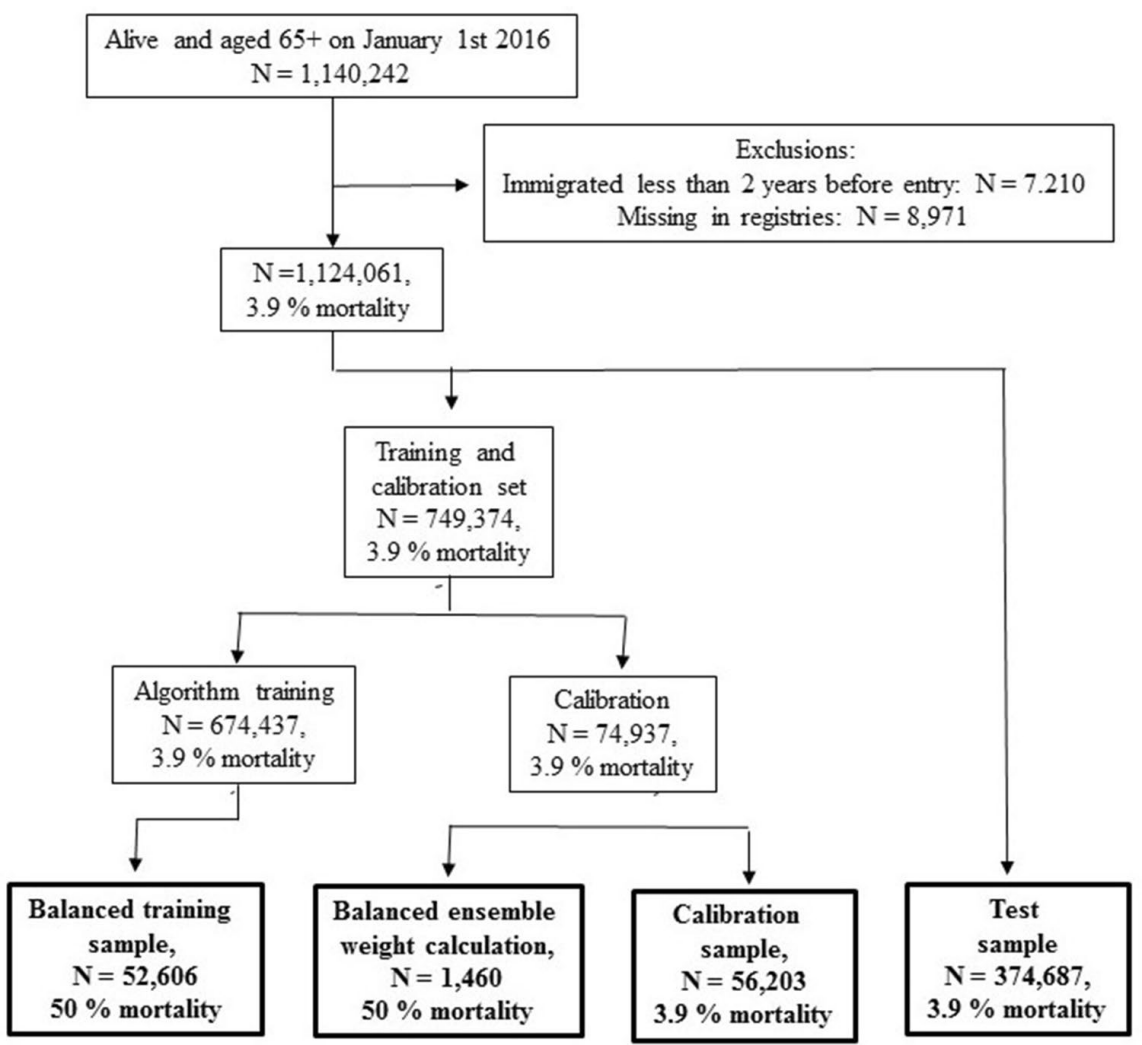
Chart describing inclusion in the study population, and data flow into the prediction algorithm. Individuals were included in the national population study if they were alive and aged 65 + on January 1st 2016 and had been living in Denmark for two years prior to this baseline date. A random sample of two thirds was selected, and a prediction model was trained on this sample, predicting individual-level mortality risk in the year after baseline based on characteristics observed during the two years before baseline. The prediction model was then used to predict one-year mortality risk for the remaining test sample, and this sample was followed up for mortality and healthcare expenditure for up to one year after baseline.

We identified 1,140,242 Danish residents aged 65 and above on Jan 1st 2016 (Fig. 1). After exclusions of 7210 who had immigrated less than two years before start of follow-up and 8971 who were missing from registries, we were left with a study population of 1,124,061 of whom 43,838 (3.9%) died during the year of follow-up. The characteristics of the test population by one-year survival status is shown in Table 1.

**Table 1:** Characteristics of the test sample used for calculating health care expenditures by predicted mortality.

	Survivor		Decedent	
	360,074		14,613	
Age (n, %)				
64–69	131,478	37	1783	12
70–74	94,516	26	2104	14
75–79	61,630	17	2340	16
80–84	38,731	11	2663	18
85–89	22,210	6	2713	19
90–94	9129	3	2075	14
95–99	2143	1	786	5
100 +	237	0	149	1
Sex (n, %)				
Male	164,695	46	7018	48
Female	195,379	54	7595	52
Danish (n, %)				
Danish	343,634	95	14,043	96
Immigrant	16,440	5	570	4
Education (n, %)				
Elementary shool	132,303	37	6950	48
High school, vocational training	141,269	39	4570	31
Further education	78,135	22	1945	13
Unspecified	8367	2	1148	8
Household type (n, %)				
Cohabiting/Married	218,538	61	4864	33
Divorced	40,764	11	1865	13
Single	25,564	7	1817	12
Widow/er	75,208	21	6067	42
Multimorbidity (n, %)				
0	50,591	14	397	3
1–2	120,201	33	2,130	15
3–4	92,664	26	3,666	25
5 +	96,618	27	8,420	58
Received care services				
No	335,932	93	8638	59
(n, %)				
Home care only	18,130	5	3693	25
Nursing home	6012	2	2282	16
Predicted 1-year mortality (mean, IQR)	0.03 (0.01–0.03)		0.21 (0.06–0.34)	

**Figure 2:**
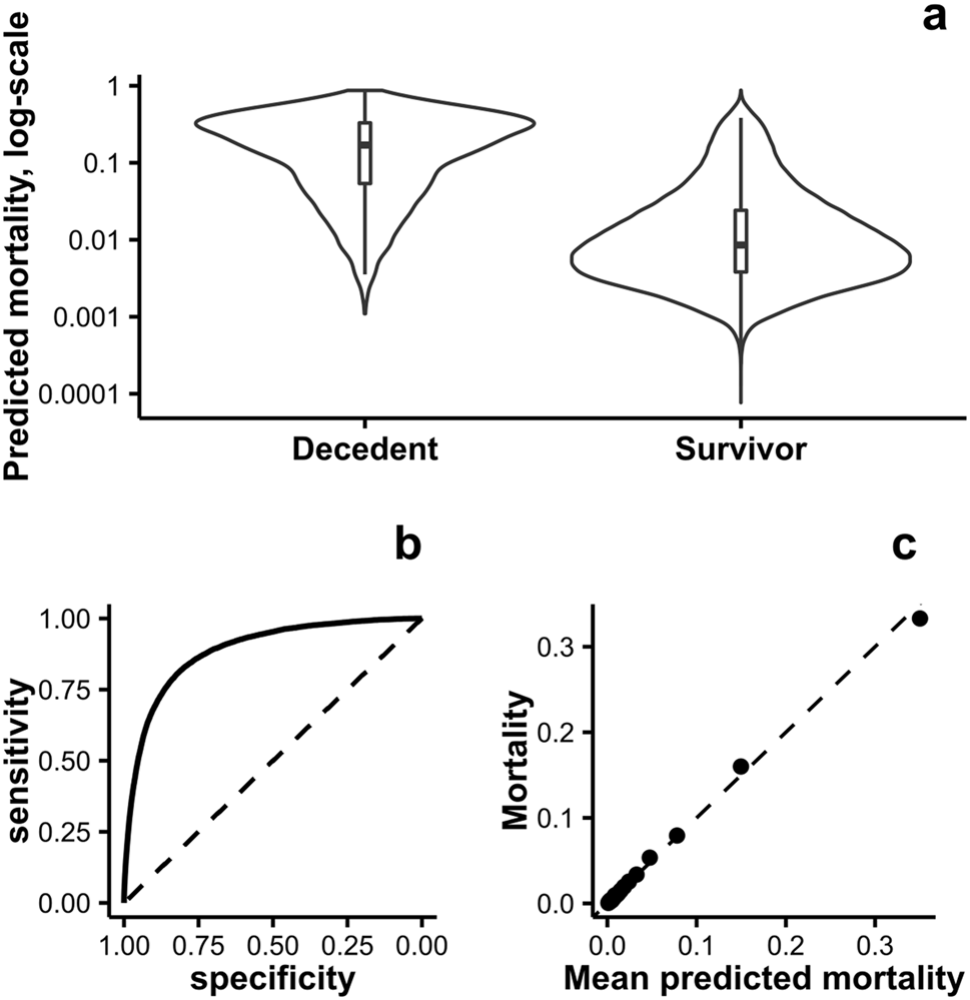
Description of the one-year mortality predictions for the test sample. Violin plot of the distribution of predicted mortalities for survivors and decedents (**a**), ROC curve (**b**), calibration plot (**c**).

Using a machine learning ensemble, the distribution of predicted one-year mortality on the test sample of 374,687 is shown in Fig. 2. While the classification was reasonably good (AUC 0.87), and the distributions of predicted mortalities for survivors and decedents were markedly distinct, only a small proportion (0.6%) had a predicted one-year mortality risk of more than 50%.

**Figure 3:**
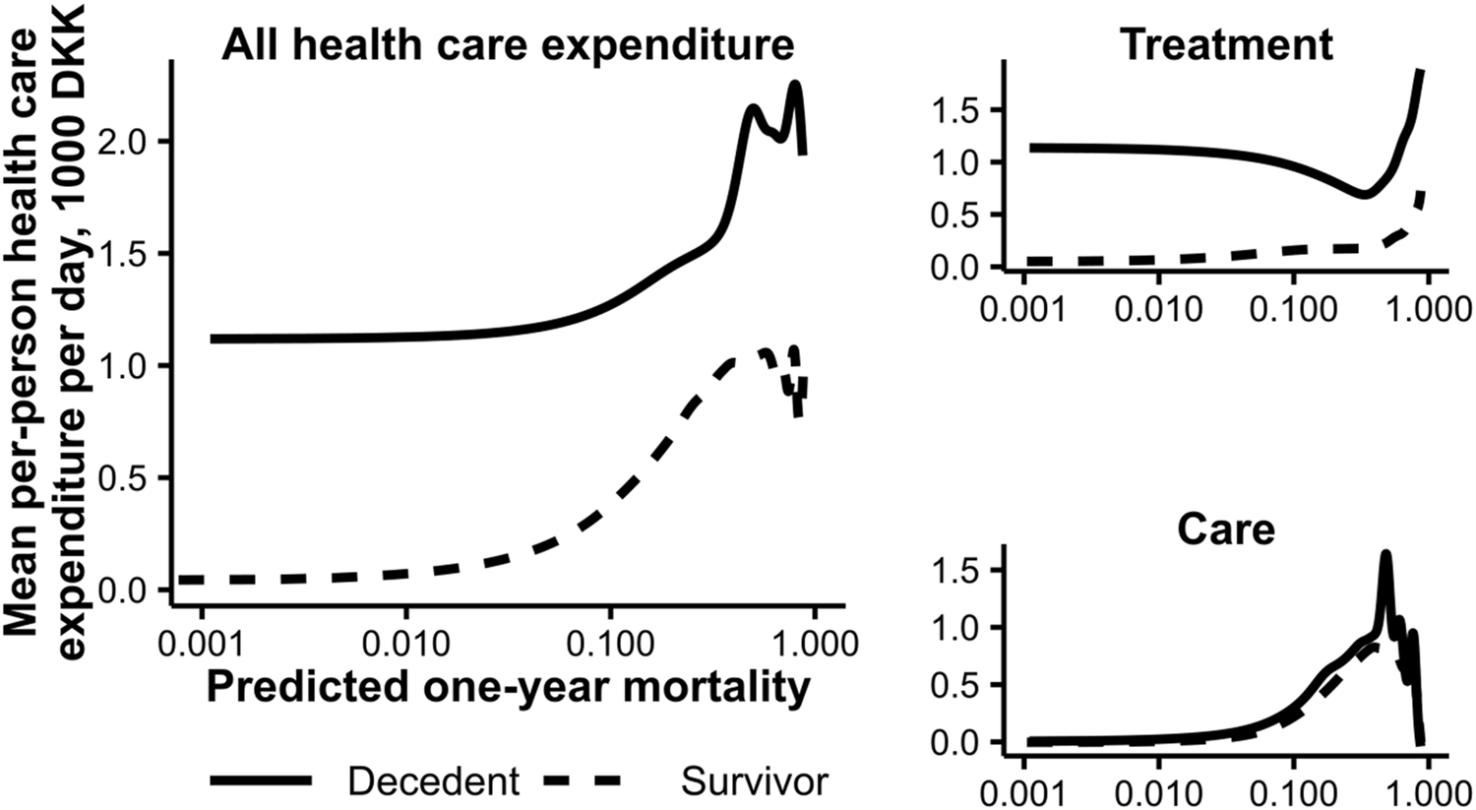
Kernel smoothed per-person healthcare expenditure per day by predicted one-year mortality and type of expenditure.

Decedents accounted for 13% of 2016 health care expenditure in the test sample (14% of care-related expenditures and 13% of treatment-related expenditures). Figure 3 shows kernel density smoothed mean individual healthcare expenditure per day alive by predicted one-year mortality for decedents and survivors and for total healthcare expenditure, treatment-related expenditure and care-related expenditure. Mean health care expenditure per day alive was higher in decedents than in survivors (1.46 and 0.14 thousand DKK per day respectively). As frail people are more likely to die and also have higher healthcare expenditures, we calculated health care expenditures in a hypothetical population of survivors with the same distribution of predicted mortality as the decedent population had. Mean health care expenditure per day alive in this hypothetical population was 0.57 thousand DKK per day, meaning that decedents outspent survivors by a factor of 10, but outspent equally frail survivors only by a factor of 2.5. Some 39 percent of decedent healthcare expenditure was explained by high spending on those with high ex-ante mortality. The corresponding percentages for treatment- and care-related expenditure, respectively, were 18% and 75%.

**Figure 4:**
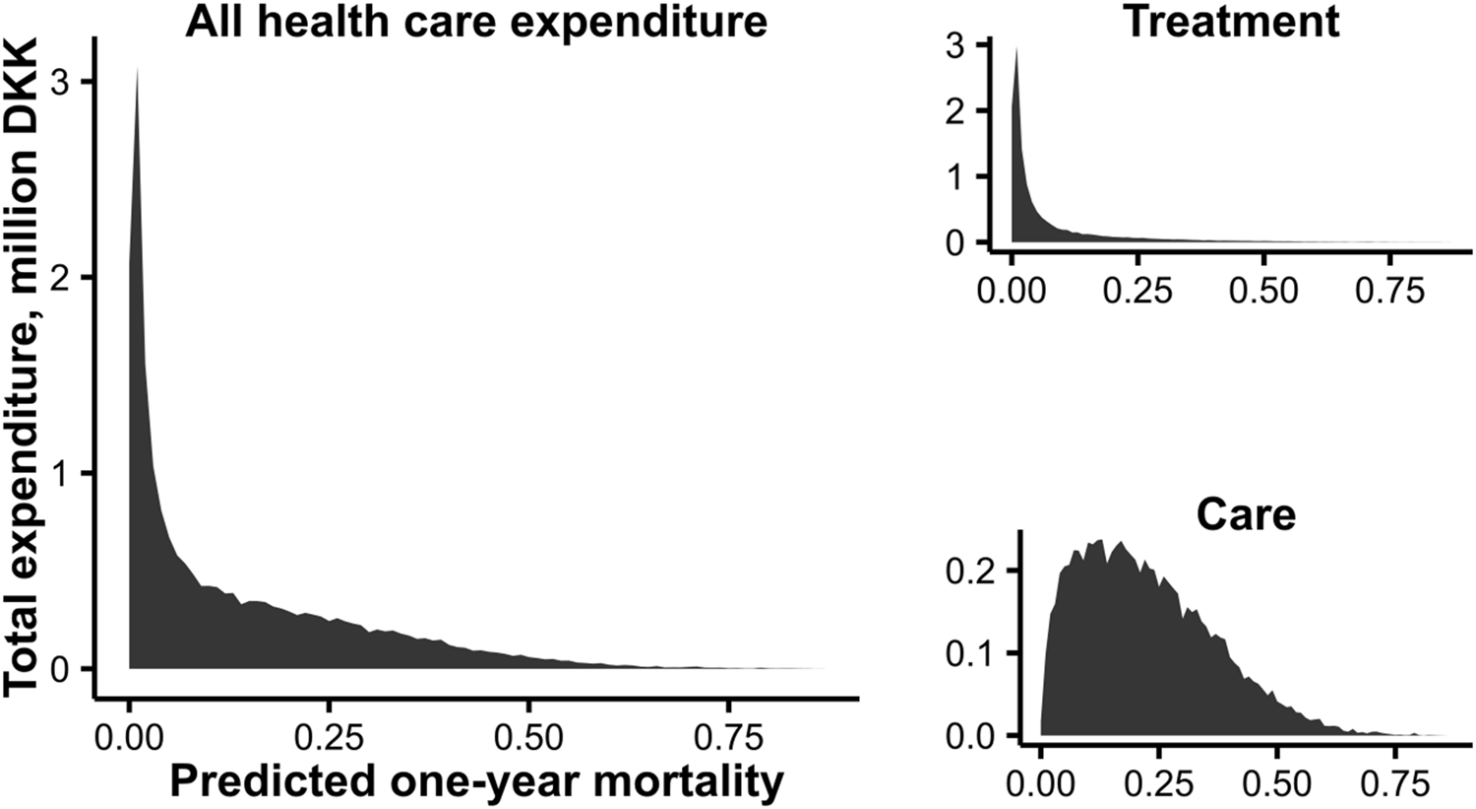
Healthcare expenditure by predicted one-year mortality expenditure and type of expenditure.

Figure 4 shows total healthcare expenditure by predicted mortality. While spending was clearly higher at higher predicted mortalities, the group with a predicted mortality of more than 50% only accounted for 2.8% of total spending. The numbers for treatment and care were 1.8% and 4.4% respectively. While 75% of total treatment-related spending occurred at a predicted mortality of less than eight percent, care-related expenditure was concentrated among those with moderately high predicted mortality, with 75% of expenditure concentrated at predicted mortalities over 11 percent.

## Discussion

Decedents accounted for 13% of yearly healthcare expenditure at age 65 and above, but only 2.8% was spent on those who, according to our machine learning model, had a likelihood of dying of more than 50%. While the mean healthcare expenditure per day alive on a decedent was ten times that of a survivor, when comparing to an equally frail population of survivors, the mean expenditure per day alive on a decedent was only 2.5 times higher. The main strength of the study is the availability of data for an entire population, with rich health care and sociodemographic predictor data and registry coverage of 97% of all healthcare expenditure^12^, as well as the inclusion of communal care in addition to treatment. As healthcare expenditure in Denmark is tax-funded, differences will not be artefacts of differential insurance coverage and rates. Individual-level expenditure data, however, may be misestimated to some extent: Hospital costs are DRG rates which are averages and may not entirely correspond to the actual cost of treatment, and the computation of individual-level expenditure on nursing home and home care involve some amount of estimation and imputation. The study deals only with expected mortality at baseline, which may arguably be a limited indicator of cost-efficiency of healthcare spending, and other measurements such as quality-adjusted life years could have been taken into account.

The distribution of predicted mortalities resemble that estimated^2^ for American Medicare enrollees. The inclusion of a wider array of personal characteristics has not improved prediction materially, as our AUC is essentially the same as that of the Medicare study—a result that compares reasonably well to what other studies have achieved^6,7,13–16^, particularly considering the relatively wide time horizon of prediction for our study. The very low proportion with high predicted mortality might be due to essential randomness in mortality, the accrual of health-impacting events after start of follow-up, or due to shortcomings of the data available. But while we absolutely might point to health indicators that were not available for the study there are indications^10,17,18^ that these may not improve mortality prediction that much.

The mass of treatment costs is concentrated at low predicted mortalities in a pattern resembling that of Einav et al.^2^. Care-related costs, conversely, are concentrated at higher mortalities and increase more markedly with increasing mortality, whereas the costs of treatment among decedents actually decrease up to a predicted mortality of about 30%. This is not surprising—predicted mortality is a proxy for frailty and thus for the need for communal care, and the need for care is likely to change less as the result of health-impacting events over the course of follow-up. It is interesting that we see a decline with predicted mortality in treatment-related expenditure per day alive for decedents. This was not observed for the American population and may reflect different medical culture in Denmark and the US, but the different prediction algorithms might also be part of the explanation—treatment-related expenditure decreases with age in Danish decedents^11^, and if a high predicted mortality is more reflective of age and frailty in our algorithm than for the American data, that might explain the difference.

At similar predicted mortalities, there is little difference between the care-related expenditure per day alive of decedents and survivors. The treatment-related expenditure of decedents is much higher than that of survivors, although the differences are lower at higher mortalities. This pattern may in part be explained by the passage of time—by the time a person dies, their health has likely deteriorated since their status at entry, and it seems likely that a person who dies at low predicted mortality will have experienced some dramatic health event requiring treatment, while death at higher predicted mortality might be a more direct continuation of patterns already established by the time of entry. Also, a person with low predicted mortality might be a better candidate for treatment, being less frail. But to the extent that the difference between survivors and decedents at the same mortality is not due to curveball events, it might be seen as the “true” cost of dying.

Thus, nearly all healthcare expenditure occurs in situations where there is a reasonable expectation that the patient can survive, and so the concept of “the cost of dying” is confounded by frailty: We spend more on the frail, and the frail are more likely to die—but not certain to do so, at least within a relevant time frame. This underlying frailty, operationalized as high predicted one-year mortality accounted for 39% healthcare expenditure in the last year of life in Denmark, an estimate in line with that in American Medicare enrollees^2^. The idea of a potential for reductions in health care expenditure at the end of life is enticing, and it seems possible to find groups that could benefit from a switch to a palliative course of treatment. Still, our results, along with those of our model paper, add to a list of arguments for why it might be illusory to reduce healthcare expenditure much by cutting the cost of dying. The proportion of spending occurring at the end of life is lower than has previously been reported^1^, decedents make up a relatively small share of high-cost individuals^3^, rising levels of demand drive increasing health care costs in ageing populations at least as much as the cost of dying^19^, and high end-of-life costs seem driven more by multimorbidity than last-ditch lifesaving efforts^1,11,20^. Our study design does not touch upon the question of individual treatment effects—whether specific treatments improve survival for specific individuals—and it may be that better methods than ours can detect high-mortality subgroups, but it seems unlikely for such subgroups to be large enough that costs reductions there could matter on the scale of a national budget.

## Methods

The use of personal data in this study followed Danish data protection legislation. Treatment of the data at Statistics Denmark is legal according to the Act on Statistics Denmark^21^ and the General Data Protection Regulation (GDPR) art 6 s 1 ss e^22^, Statistics Denmark has the right to process individual-level registry data. Per GDPR art 14 s 5 ss b^22^, obtaining individual consent for the use of registry data in research is not required.

The study design is illustrated in Fig. 1. The study population consisted of all individuals of age 65 and above who had been living in Denmark for two years at the point of entry into the study (January 1st 2016). We collected personal characteristics from individually linkable national registries for the last two years before entry and followed up for death or emigration for one year after entry, as well as for use of healthcare services in the follow-up period (Supplementary information 1).

A machine-learning algorithm to predict one-year survival was trained on a training sample consisting of two thirds of the population. The algorithm was an ensemble of three methods: Boosting, Random Forest and Lasso. Sub-samples of 2.5% and 7.5% of the training sample were set aside for ensemble weight calculation and calibration, the remaining training sample was reduced to a balanced sample with a 50–50 distribution of decedents and survivors by random selection of a subsample of survivors, and the three learners were trained on the balanced training sample, with parameter tuning by five-fold cross-validation. Having trained the individual learners, we created an ensemble by weighting them together, using the ensemble weight calculation sample (after reducing this to a balanced sample) to compute weights by computing predicted values for the three learners and fitting a linear combination of these to the observed values. As the prediction ensemble was trained on balanced data, we re-balanced it, using Bayes’ rule. Following our model paper, we would have used the calibration sample to fit a third-degree polynomial in the predicted values to observed mortality, but as we observed better calibration simply using Bayes’ rule, we abandoned that approach. The prediction model was trained using R software and the tidymodels framework^23^, and the ranger^24^, xgboost^25,26^ and glmnet^27^ packages.

The personal characteristics were sex, age, country of origin, education, marital status and household size, municipality, variables on income and financial assets, use of primary healthcare grouped by specialization, number of hospital contacts by ICD-10 chapter of the main diagnosis for the contact, number of prescriptions by five-digit ATC code, amount of home care (personal and practical help) being provided by the municipality and an indicator for admission to a nursing home. The financial variables were collected on a yearly basis for the last two years before entry, all other time-varying variables were collected on a quarterly basis for the last two years before entry. In order to reduce the size of the predictor space, we performed principal component analysis on the quarterly-level prescription data and reduced to the first 66 components.

The trained algorithm was used to predict one-year survival for the test sample of one third of the population, and the distribution of healthcare expenditure over the year of follow-up by predicted mortality at one year was examined. Healthcare expenditure was defined either treatment-related: The costs of hospitals, primary care and prescription drugs; or care-related: The costs of home care and residential care. Healthcare and eldercare in Denmark is primarily taxpayer-funded, so the information available in registries accounts for 97% of healthcare expenditure in Denmark^12^. Individual-level expenditures for the period of follow-up were computed, as well as individual-level expenditure per day of follow-up, for total health expenditure and for treatment- and care-related expenditures. The methods are described in^11^.

In order to assess the proportion of health care expenditure explained by predicted mortality, we reweighted the survivor population to have a similar distribution of predicted mortality as the decedent population and compared mean spending per day alive in the weighted survivor population to mean spending among decedents. The weights were computed, using kernel density estimates of the densities among decedents and survivors with a survivor with an estimated survival probability of p being given a weight of k_d_(p)/k_s_(p) where k_d_ is the density function estimated for decedents and k_s_is the density function estimated for survivors.

## Data availability statement

Due to restrictions in Danish law, the confidential health care data used in this study can only be accessed through Statistics Denmark, the state organization holding the rights to the data. Danish scientific organizations can be authorized to work with data within Statistics Denmark and can provide access to individual scientists inside and outside of Denmark. Data are available via the Research Service Department at Statistics Denmark: (www.dst.dk/da/TilSalg/Forskningsservice) for researchers who meet the criteria for access to confidential data. The authors of this study had no special access privileges others would not have.

## Supplementary Information

Supplementary Information.
