# Supplementary material for: Predicting mortality and visualizing health care spending by predicted mortality in Danes over age 65
Source: Sci Rep. 2023 Jan 21;13:1203. doi: 10.1038/s41598-023-28102-4 (PMC9867694; doi:10.1038/s41598-023-28102-4)
Supplement: Supplementary file 1 — Supplementary Information. [file 41598_2023_28102_MOESM1_ESM.docx]

| **Predictors** | | **Source** |
| --- | --- | --- |
| **Predictors constant at the individual level** | |  |
|  | Age by Jan 1^st^ 2016, Sex, Immigrant status, Country of origin | Statistics Denmark population registry [1] |
|  | Highest attained level of education | The Population’s Education Register [2] |
|  | Municipality of residence by Jan 1^st^ 2016 | Statistics Denmark population registry [1] |
|  | Length of stay for residents in nursing home by Jan 1^st^ 2016 | Statistics Denmark data on social services for the elderly [3] |
|  | Affluence index [4] computed at age 65 | Income Statistics Register [5] |
| **For each quarter of 2014-2015:** | |  |
|  | Civil status, cohabitation status, household type, number of persons in household/family | Statistics Denmark population registry [1] |
|  | Hours of personal and practical care provided by municipality | Statistics Denmark data on social services for the elderly [3] |
|  | Number of hospital contacts by ICD-10-chapter of main diagnosis | Danish National Patient Register [6] |
|  | Number of primary care visits by type of treatment | Danish National Health Service Register [7] |
|  | Number of prescriptions by ATC-code | Danish National Prescription Register |
| **Yearly, for the years 2011-2015:** | |  |
|  | Total salary, disposable income, amount of national early pension, amount of private pension, amount of state benefits, amount of property income | Income Statistics Register [5] |

1. Statistikdokumentation: Befolkningen. at <https://www.dst.dk/da/Statistik/dokumentation/statistikdokumentation/befolkningen>

2. Jensen, V. M. & Rasmussen, A. W. Danish education registers. *Scand. J. Public Health* **39,** 91–94 (2011).

3. Statistikdokumentation: Sociale ydelser til ældre. at <https://www.dst.dk/da/Statistik/dokumentation/statistikdokumentation/sociale-ydelser-til-aeldre>

4. Cairns, A. J. G., Kallestrup Lamb, M., Rosenskjold, C., Blake, D. P. & Dowd, K. *Modelling Socio-Economic Differences in Mortality Using a New Affluence Index*. (Social Science Research Network, 2019). doi:10.2139/ssrn.3376527

5. Baadsgaard, M. & Quitzau, J. Danish registers on personal income and transfer payments. *Scand. J. Public Health* **39,** 103–105 (2011).

6. Lynge, E., Sandegaard, J. L. & Rebolj, M. The Danish National Patient Register. *Scand. J. Public Health* **39,** 30–33 (2011).

7. Andersen, J. S., Olivarius, N. D. F. & Krasnik, A. The Danish National Health Service Register. *Scand. J. Public Health* **39,** 34–37 (2011).
